# Expression profiles during dedifferentiation in newt lens regeneration revealed by expressed sequence tags

**Published:** 2010-01-18

**Authors:** Nobuyasu Maki, John Martinson, Osamu Nishimura, Hiroshi Tarui, Jaroslaw Meller, Panagiotis A. Tsonis, Kiyokazu Agata

**Affiliations:** 1 Center for Developmental Biology, RIKEN Kobe, Kobe, Japan; 2 Department of Biology and Center for Tissue Regeneration and Engineering, University of Dayton, Dayton, OH; 3 Department of Biomedical Engineering, University of Cincinnati, Cincinnati, OH; 4 Ecological Exposure Research Division, Cincinnati, OH; 5 Department of Environmental Health, University of Cincinnati, Cincinnati, OH; 6 Department of Biophysics, Graduate School of Science, Kyoto University, Kyoto, Japan

## Abstract

**Purpose:**

The adult newt can regenerate lens from pigmented epithelial cells (PECs) of the dorsal iris via dedifferentiation. The purpose of this research is to obtain sequence resources for a newt lens regeneration study and to obtain insights of dedifferentiation at the molecular level.

**Methods:**

mRNA was purified from iris during dedifferentiation and its cDNA library was constructed. From the cDNA library 10,449 clones were sequenced and analyzed.

**Results:**

From 10,449 reads, 780 contigs and 1,666 singlets were annotated. The presence of several cancer- and apoptosis-related genes during newt dedifferentiation was revealed. Moreover, several candidate genes, which might participate in reprogramming during dedifferentiation, were also found.

**Conclusions:**

The expression of cancer- and apoptosis-related genes could be hallmarks during dedifferentiation. The expression sequence tag (EST) resource is useful for the future study of newt dedifferentiation, and the sequence information is available in GenBank (accession numbers; FS290155-FS300559).

## Introduction

Some species are endowed with unique physiology and provide new paradigms for both basic and applied biology. For example, adult newts can regenerate body parts, including lens, limb, tail, jaw, small intestine, brain, and heart. This regenerative ability is the highest even among amphibians, including axolotls and frogs [1,2]. It is well known that lens regeneration is mediated by dedifferentiation and transdifferentiation of terminally differentiated pigmented epithelial cells (PECs). After lens removal, PECs in dorsal irises undergo dedifferentiation where PECs exclude pigment granules and lose their cellular identity, proliferate, and then differentiate into lens cells. Although lens regeneration never occurs from the ventral iris, dedifferentiation events, depigmentation, proliferation, and gene expression are observed in ventral iris PECs [2]. Transdifferentiation of PECs has been directly demonstrated by clonal culture experiments [3,4].

For cells to change their identity and assume a new fate, a considerable degree of gene regulation must take place. It has been shown that nucleostemin, a stem cell-specific nucleolar protein found in mammals [5], accumulates in nucleoli as PECs dedifferentiate during lens regeneration [6]. We have also recently reported that mammalian stem cell pluripotency-maintaining factors cellular myelocytomatosis oncogene, sex determining region Y box 2, and kruppel-like factor 4 are expressed and regulated during lens regeneration as well [7]. These data suggest that a PEC is reprogrammed to a stem cell-like cell during dedifferentiation. However, information about molecular events during dedifferentiation is not well elucidated.

To understand the process of dedifferentiation, analysis of global gene expression during dedifferentiation is needed. Although more than 34,000 cDNA sequences for the axolotl *Ambystoma mexicanum*, 677,000 cDNAs for the African clawed frog *Xenopus laevis*, and 1,271,000 cDNAs for the western clawed frog *Xenopus tropicalis* are available [8-13], cDNA resources are lacking in the newt field. Here, we generated expression sequence tags (ESTs; 1,368 contigs and 3,357 singlets) from the iris undergoing dedifferentiation during the process of lens regeneration and analyzed their expression profiles.

## Methods

### Animals

One hundred Japanese newts, *Cynops* *pyrrhogaster*, were collected in the northern part of Okayama prefecture. All animal procedures were approved by animal care board in Center for Developmental Biology, Riken Kobe. Newts were euthanized by anesthesia (soaking in 0.1% of MS-222 [Sigma-Aldrich, Tokyo, Japan] for 15 min) followed by decapitation.

### mRNA extraction

Both dorsal and ventral irises 8 days after lentectomy (when dedifferentiation events of PECs are ongoing, i.e., initiation of depigmentation and proliferation [6]) were collected for the cDNA library of lens-regenerating iris. mRNA was purified using Dynabeads Oligo(dT)_25_ (Dynal Biotech, Oslo, Norway) according to the manufacturer’s instructions. Briefly, Dynabeads Oligo (dT) was added to homogenized iris sample. mRNA hybridized to Dynabeads Oligo(dT) was isolated by magnetic separation, washed, and eluted by incubation with 10 mM Tris (2-amino-2-hydroxymethylpropane-1,3-diol)-HCl buffer, pH 7.5 at 75 ºC for 2 min.

### Construction of the cDNA library

The cDNA library was constructed using the ZAP-cDNA synthesis kit (Stratagene Japan, Kunitachi, Japan). Reverse transcription reaction was performed using oligo-dT primer, and the synthesized cDNAs were directionally inserted into a Uni-ZAP XR Vector (Stratagene Japan). The vector-containing cDNA was packaged in a lambda phage, using a Gigapack III gold cloning kit (Stratagene Japan). pBluescript phagemid was prepared by in vivo excision using ExAssist helper phage  (Stratagene Japan) and XLI-Blue MRF strain (Stratagene Japan). XLI-Blue MRF strain cells were transformed with the phagemid and plated on L-Broth-ampicilin plate.

### Sequencing of cDNA

Each colony was picked using an automated colony picker Qpix (Genetix K.K., Toyo, Japan), and template DNA for sequencing was amplified using the TempliPhi DNA sequencing template amplification kit (GE Healthcare Life Sciences, Piscataway, NJ). The sequencing reaction was performed using the BigDye terminator v. 3.1 cycle sequencing kit (Applied Biosystems, Foster City, CA). The primer used in the sequence reaction was the T3 primer (Stratagene Japan). The sequence reaction products were analyzed using a 3730xl DNA analyzer (Applied Biosystems). The GenBank accession numbers for ESTs are FS290155-FS300559.

### Assembly of sequence data

To remove possible low-quality fragments at both edges of individual reads, 20 bases were trimmed off each end of all sequences. Then, potential vector contamination was removed or masked. Individual reads from all tissues were assembled using CAP3 software with default settings [14].

### Functional annotation

Functional annotation of the assembled sequences was performed using the Blast2GO program with default settings [15]. Putative “gene names” and functions are also assigned to sequences with GO annotations as part of the annotation process. The annotations were augmented by the Annotation Expander (ANNEX) software [16].

## Results and Discussion

### Sequencing, assembly, and functional annotation of newt expression sequence tags

As mentioned previously dedifferentiation events occur in the ventral iris as well as the dorsal iris. Therefore dorsal and ventral irises 8 days after lentectomy, when dedifferentiation of PECs is ongoing, were collected, cDNA libraries were constructed using mRNA from the regenerating iris, and ESTs from the iris libraries were obtained. The initial set of cDNA sequence reads were 10,449 (Table 1). The total length of these sequences was 9.85 Mb, which was reduced to 4.82 Mb by assembling overlapping reads, using CAP3 software. This assembly was composed of 4,725 unique sequences (1,368 contigs and 3,357 singlets). Of 1,219 contigs and 3,357 singlets, 780 contigs and 1,666 singlets were annotated by Blast2GO.

**Table 1:** Process of lens-regenerating iris ests assembly into contigs.

**Assembly**	**Number of sequences**	**Number of contigs**	**Number of singlets**	**Average contig length (bp)**	**Average singlet length (bp)**	**Total length of sequences (Mbp)**
Initial reads after Phred analysis	10,449				943	9.85
After trimming 20 bases from ends and vector screening	10,405				899	9.35
After cap3 assembly	4,725	1,368	3,357	1,279	915	4.82

### Expression profiles of iris ongoing dedifferentiation

**Figure 1:**
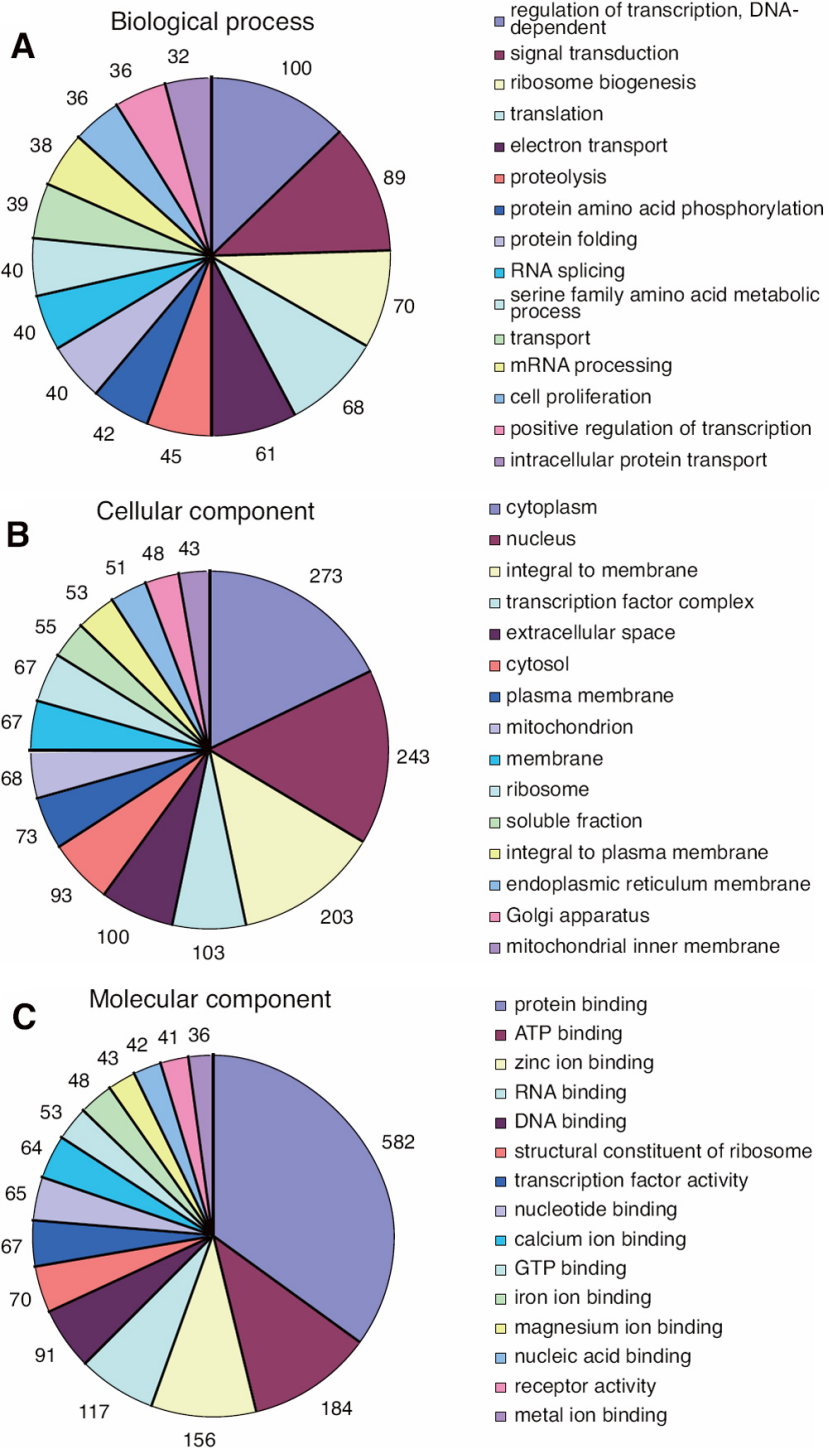
Most frequently counted terms in the lens-regenerating iris. The fifteen most frequently counted terms of biologic process (**A**), cellular component (**B**), and molecular function (**C**) are shown. The total number of these most frequently counted terms was 5,356, 3,259, and 4,152, respectively. In the figure, ATP is adenosine triphosphate and GTP is guanosine triphosphate

Functional annotation of the contigs and singlets was performed using the Blast2GO program. The 15 most frequently counted biologic process terms, cellular component terms, and molecular function terms are shown in Figure 1.

Tyrosinase, a possible marker for differentiated PECs, was not found in the EST. No detection of tyrosinase in this EST might reflect changing of the original PEC gene expression 8 days after lentectomy. Nucleostemin, a nucleolar protein mainly expressed in stem cell populations in mammals [5], was found in the ESTs (Table 2). This result was consistent with a previous report in which nucleostemin emerged in nucleoli during newt dedifferentiation [6]. Crystallins, which are known to be lens differentiation markers, were not found in the EST list, suggesting that these ESTs consist of cDNAs involved in the dedifferentiation stage before lens differentiation.

**Table 2:** Apoptosis related genes expressed during dedifferentiation.

**Annotation of sequence**
Apoptosis-inducing mitochondrion-associated 1
BCL2-like 13
BCL2-associated agonist of cell death
Programmed cell death 2
Programmed cell death 4
Programmed cell death 5
Programmed cell death 6
Programmed cell death 6 interacting protein

All the sequences shown have e-value less than 3.5E-17.

A close examination of the list revealed that several members of the bone morphogenetic protein and transforming growth factor pathways were present in our ESTs. We were unable to find any from the wingless-type mouse mammary tumor virus integration site family, fibroblast growth factor, or sonic hedgehog pathway. The reason for the presence or absence could be due to the stage of the iris that was isolated. The iris was at the dedifferentiation stage. It is possible that pathways that are important for lens differentiation, such as fibroblast growth factor or sonic hedgehog, are simply not active at that stage [17,18]. Alternatively, it is possible that certain factors were not cloned in our experiment.

It appears that a wide variety of cancer-related genes were expressed during dedifferentiation (Table 3). A total of 27 cancer-related genes were found in the ESTs, especially a large number of Ras-related genes and tumor necrosis factor-related genes. The expression of cancer-related genes might be related to the initiation of proliferation during dedifferentiation. Additionally, as c-Myc is one of four factors (cellular myelocytomatosis oncogene, octamer-binding transcription factor 4, sex determining region Y box 2  and kruppel-like factor 4) to reprogram somatic cells to pluripotent stem cells [19], there is a possibility that cancer-related genes play a role in reprogramming during dedifferentiation. In addition to cancer-related genes, eight apoptosis-related genes were found in the EST list (Table 2). The expression of cancer- and apoptosis-related genes could be one of the hallmarks during dedifferentiation in newt regeneration.

**Table 3:** Cancer-related genes found in iris during dedifferentiation.

**Annotation of sequence**	**Annotation of sequence**
**Ras**	**Tumor necrosis factor**
Kras	Tumor necrosis factor alpha
Ras associated protein RAB1	Tumor necrosis factor alpha-induced protein 6
Ras homolog gene family, member T1	Tumor necrosis factor receptor associated factor 2
Ras homolog gene member a	Tumor necrosis factor receptor member 1b
Ras p21 protein activator 4 isoform 1	Tumor necrosis factor receptor member 14
Ras related GTP-binding protein B	
Ras related nuclear protein	**Tumor protein**
	Tumor protein d52
**Retinoblastoma**	Tumor protein d52-like 2
Retinoblastoma binding protein 4 isoform 1	
	**Others**
**Jun**	Fat tumor supressor, homolog 1
c-jun	Feline sarcoma oncogene
Jun-B oncogene	Fyn oncogene related to yes*
	Glioma tumor suppressor candidate region gene 2
**p53**	Large tumor supressor, homolog 1
Tumor protein p53	Leydig cell tumor 10 kDa protein
p53-associated parkin-like cytoplasmic protein	Tumor translationally-controlled 1

All the sequences shown have e-value less than 8.2E-16.

### Candidate genes that regulate reprogramming during dedifferentiation

From our list of the lens-regenerating iris ESTs, possible candidate genes for participating in nuclear regulation during newt dedifferentiation were identified (Table 4). Epigenetic regulation, a range of heritable chromatin modifications, including histone modifications, DNA methylation, and chromatin remodeling, play a pivotal role in the control of differentiation and maintenance of cellular identity. It is therefore expected that epigenetic regulation plays an important role during newt dedifferentiation.

**Table 4:** Candidate genes which regulate reprogramming during dedifferentiation.

**Annotation of sequence**	**Annotation of sequence**
**Histone acetyltransferase**	**Non-histone chromosomal protein**
CREB binding protein / p300*	High-mobility group protein box 2*
Histone acetyltransferase MYST3*	High-mobility group protein box 3
	
**Histone deacetylase**	**Nucleolar protein**
Histone deacetylase 2*	Nucleostemin*
Histone deacetylase 5*	
	**Transcriptional factor/repressor**
**Histone demethylase**	Nf-kappaB*
Jumonji domain containing 1b	COUP transcription factor 1*
Jumonji domain containing 2a*	REST/RE1-silencing transcription factor*
	
**DNA methyltransferase**	
DNA (cytosine-5-)-methyltransferase 1*	
Williams beuren syndrome chromosome region 22*	

All the sequences shown have e-value less than 1.0E-35. The asterisk indicates genes commonly found in ovary ESTs but not in intact limb ESTs [unpublished data].

Histone acetylation is generally related to transcriptional activation and mediated by histone acetyltransferase. In the ESTs, there were two types of histone acetyltransferases, cyclic adenosine monophosphate response element-biding protein binding protein/p300 [20] and MYST3 [21], as well as two deacetylases, histone deacetylase 2 and 5 (HDAC2, HDAC5; Table 4) [22]. The balance between histone acetyltransferase and deacetylase might be strictly regulated during newt dedifferentiation.

To form heterochromatin, heterochromatin protein 1 is recruited to methylated histone H3K9 [23,24]. It has been demonstrated that the Jumonji domain-containing 2a (JMJD2A) is a histone demethylase against histone H3K9 and H3K36 and antagonizes heterochromatin formation via histone H3K9 [25]. It is thought that JMJD1B is a H3K9 histone demethylase because JMJD1B has a JmjC domain [26]. Interestingly, JMJD2A and JMJD1B were found in the ESTs (Table 4). This might indicate that these histone demethylases eliminate heterochromatin during dedifferentiation.

DNA methylation is a covalent modification of DNA and confers a heritable gene repression during and after development [27,28]. DNA (cytosine-5-)-methyltransferase 1 [28] and putative DNA methyltransferase Williams Beuren syndrome chromosome region 22 [29] were found in the ESTs (Table 4).

The high mobility group protein is a nonhistone chromatin protein. In vitro experiments have demonstrated that high mobility group protein box 2 (HMGB2) nonspecifically binds and bends DNA. It is suggested that HMGB2 facilitates cooperative interactions between cis-acting proteins by promoting DNA flexibility [30]. Like HMGB2, HMGB3 contains DNA-binding HMG box domains and is thought to be able to alter DNA structure [31]. Thus, HMGB2 and HMGB3 might promote genome-wide DNA flexing, which allows new sets of gene expression during dedifferentiation.

Transcriptional factors, nuclear factor-κB [32], and chicken ovalbumin upstream promoter transcription factor 1 [33] were found. Repressor element 1 silencing transcriptional factor (REST) binds to repressor element 1 and recruits a wide variety of chromatin modification enzymes, such as the histone deacetylases HDAC1 and HDAC2, histone H3K9 methylases G9a and SUV39H1, and a histone H3K4 demethylase lysine-specific demethylase 1 (LSD1) directly or indirectly with the CoREST complex or the mammalian switch independent 3 (mSin3) complex [34]. Interestingly, REST/RE1-silencing transcription factor and HDAC2 were found in the ESTs, suggesting cooperation of these molecules during dedifferentiation.

The oocyte has an ability to reprogram the somatic nucleus, which was demonstrated by the nuclear transfer into an oocyte [35]. Interestingly, most of the nuclear genes identified as candidates to regulate newt dedifferentiation were found in ovary ESTs as well (14,429 reads) but not in intact limb ESTs (1,098 reads; Table 4). Functional analysis of these genes might provide an advanced understanding of cellular plasticity, with possible future applications in regenerative therapies.
